# Identifying *SYNE1* Ataxia With Novel Mutations in a Chinese Population

**DOI:** 10.3389/fneur.2018.01111

**Published:** 2018-12-20

**Authors:** Yun Peng, Wei Ye, Zhao Chen, Huirong Peng, Puzhi Wang, Xuan Hou, Chunrong Wang, Xin Zhou, Xiaocan Hou, Tianjiao Li, Rong Qiu, Zhengmao Hu, Beisha Tang, Hong Jiang

**Affiliations:** ^1^Department of Neurology, Xiangya Hospital, Central South University, Changsha, China; ^2^School of Information Science and Engineering, Central South University, Changsha, China; ^3^Laboratory of Medical Genetics, Central South University, Changsha, China; ^4^National Clinical Research Center for Geriatric Diseases, Xiangya Hospital, Central South University, Changsha, China; ^5^Key Laboratory of Hunan Province in Neurodegenerative Disorders, Central South University, Changsha, China; ^6^Parkinson's Disease Center of Beijing Institute for Brain Disorders, Beijing, China; ^7^Collaborative Innovation Center for Brain Science, Shanghai, China; ^8^Collaborative Innovation Center for Genetics and Development, Shanghai, China; ^9^Xinjiang Medical University, Ürümqi, China

**Keywords:** cerebellar ataxia, *SYNE1*, mutation, high-throughput nucleotide sequencing, Genotype–Phenotype

## Abstract

**Objective:** Variants in *SYNE1* have been widely reported in ataxia patients in Europe, with highly variable clinical phenotype. Until now, no mutation of *SYNE1* ataxia has been reported among the Chinese population. Our aim was to screen for *SYNE1* ataxia patients in China and extend the clinicogenetic spectrum.

**Methods:** Variants in *SYNE1* were detected by high-throughput sequencing on a cohort of 126 unrelated index patients with unexplained autosomal recessive or sporadic ataxia. Pathogenicity assessments of *SYNE1* variants were interpreted according to the ACMG guidelines. Potential pathogenic variants were confirmed by Sanger sequencing. Clinical assessments were conducted by two experienced neurologists.

**Results:** Two Chinese families with variable ataxia syndrome were identified (accounting for 1.6%; 2/126), separately caused by the novel homozygous *SYNE1* mutation (NM_033071.3: c.21568C>T, p.Arg7190Ter), and compound heterozygous *SYNE1* mutation (NM_033071.3: c.18684G>A, p.Trp6228Ter; c.17944C>T, p.Arg5982Ter), characterized by motor neuron impairment, mental retardation and arthrogryposis.

**Conclusions:** *SYNE1* ataxia exists in the Chinese population, as a rare form of autosomal recessive ataxia, with a complex phenotype. Our findings expanded the ethnic, phenotypic and genetic diversity of *SYNE1* ataxia.

## Introduction

Mutations in *SYNE1* were first discovered to cause autosomal recessive cerebellar ataxia among French-Canadian pedigrees in 2007, referred to as autosomal recessive cerebellar ataxia type 1 (ARCA1), also called spinocerebellar ataxia autosomal recessive 8 (SCAR8), which is described as an adult-onset, relatively pure cerebellar ataxia (1). Due to the large size of *SYNE1*, the most giant isoform (nesprin-1 giant or enaptin) of which consists of 146 exons, it is hard to screen *SYNE1* gene by conventional Sanger sequencing in ataxia patients. Following recent progress in the development of high throughput sequencing technologies, variants in *SYNE1* have been widely reported in ataxia patients from Europe, with other cases from South America, North America, Oceania, Africa, and Asia (2–18). These reported *SYNE1* ataxia patients show a strong heterogeneity in clinical features and disease severity, ranging from a pure cerebellar ataxia to a complex multisystem disorder (19). Till now, only four Japanese and two Korean *SYNE1* ataxia families were reported in Eastern Asia population, and no mutation of *SYNE1* ataxia has been reported among the Chinese population. Here, we screened *SYNE1* mutations among a cohort of 126 unrelated index patients with unexplained autosomal recessive or sporadic ataxia in China, by whole-exome sequencing or targeted panel sequencing. Our aim was to search for *SYNE1* ataxia patients in China and describe their clinical characteristics.

## Methods

### Patients

A cohort of 126 unrelated index patients (31 autosomal recessive, 95 sporadic) were enrolled from the Department of Neurology, Xiangya Hospital of Central South University from 2013 to 2017 (Supplementary Material_Table S1). The age at onset (AAO) was 31.3 ± 14.9 (mean ± SD) ranging from 5 to 59 years. They were diagnosed with unexplained autosomal recessive or sporadic ataxia, in whom repeat expansion disorders including SCA1, SCA2, SCA3, SCA6, SCA7, SCA8, SCA10, SCA12, SCA17, SCA31, SCA36, DRPLA, and Friedreich's ataxia have been excluded. Patients (*n* = 89) enrolled from January 2013 to November 2016 were screened by targeted panel sequencing, and patients enrolled from December 2016 to December 2017 were screened by whole exome sequencing (*n* = 37). The decision of change in screening strategy (from targeted panel sequencing to whole exome sequencing) were made in December 2016, due to the increasing evidence of the priority of the whole exome sequencing in undiagnosed inherited and sporadic ataxias over the targeted panel sequencing (20). This study was approved by the Ethics Committee of Xiangya Hospital of Central South University. Written informed consent was obtained from all subjects engaged in this study.

### Genetic Testing

#### DNA Preparation

Genomic DNA was extracted from EDTA-anticoagulated blood from index patients using a standard phenol–chloroform method. Additional samples were taken from affected or unaffected relatives to test for segregation analysis when necessary.

#### Whole-Exome Sequencing

Thirty-seven patients were screened for *SYNE1* mutations as part of a whole exome-sequencing study. Genomic DNA was fragmented into 250–300 bp length fragments with the use of sonication. The DNA fragments were then processed by end-repairing, A-tailing and adaptor ligation, a 4-cycle pre-capture PCR amplification, and enriched by xGen Exome Research Panel (Integrated DNA Technologies, Skokie, IL, USA). Paired-end sequencing (150 bp) was performed on Illumina HiSeq X-ten platform to provide a mean sequence coverage of more than 100 ×, with more than 95% of the target bases having at least 20 × coverage. The reads were mapped to the human reference genome (UCSC hg19) with the use of the BWA (version 0.7.15, http://bio-bwa.sourceforge.net) (21), duplicate sequence reads were removed by Picard (version 2.10.3; http://picard.sourceforge.net), and GATK (version 3.2, https://software). broadinstitute.org/gatk/was used to detect variants (22). Variants were annotated using Variant Effect Predictor (VEP) (23) and loaded into GEMINI (v0.20. 1) (24). Variants with minor allele frequency of 1% or higher in 1000 Genomes Project (1000 Genomes Phase 3), Genome Aggregation Database (gnomAD r2.0.2) (25) were excluded. Only non-synonymous variants, indels, and putative splice site variants were considered for further analysis. The nomenclature of variants based on the guidelines of the Human Genome Variation Society (HGVS), the DNA and protein changes were determined according to the *SYNE1* reference sequence (RefSeq NM_033071.3).

#### Targeted Panel Sequencing

Eighty-nine patients were screened for *SYNE1* variants as part of a targeted exon-capture strategy (Agilent SureSelect kit) coupled with multiplexing and high-throughput sequencing of 211 genes causing ataxia, hereditary spastic paraplegia and other related neurological disorders. The Agilent system consists in a capture of the genomic regions harboring the exons, based on the hybridization of complementary designed biotinylated cRNA oligonucleotides. Reads mapping, variant calling, and annotation followed the same method as described above in *Whole-exome sequencing*.

#### Inclusion of SYNE1 Variants

All the truncating *SYNE1* variants were first taken into consideration as it has been established as a main mutation type in ataxia patients. Missense variants were taken into consideration when it meet all the following criteria as Synofzik et al. (8) suggested: (i) segregated in trans with a truncating *SYNE1* variant; (ii) was absent or rare in public exome databases (dbSNP, 1000Genomes project, NHLBI ESP6500, ExAc, and gnomAD); (iii) located in highly conserved positions of the N-terminal actin-binding domain (codon 1–289); and (iv) predicted to be damaging by at least two out of three *in silico* algorithms [Mutation Taster (26); SIFT (27); and PolyPhen-2 (28)].

#### Sanger Sequencing

Sanger sequencing was performed to validate the putative pathogenic variants, allowing segregation analyses where possible (see Supplementary Material_Table S2 for primers employed).

### Clinical Investigation

For individuals with putative *SYNE1* pathogenic variants, the detailed clinical data were obtained and a clinical examination and evaluation was performed by two experienced neurologists, including the Scale for Assessment and Rating of Ataxia (SARA), Inventory of Non-ataxia Symptoms (INAS), Mini Mental State Examination (MMSE), Montreal Cognitive Assessment (MoCA), Wechsler Intelligence Scale for Children (WISC) or Wechsler Adult Intelligence Scale (WAIS) were performed according to the age of patient at examination. Laboratory tests were operated, including routine blood test, liver and kidney function test, blood glucose test, blood lipid test, ceruloplasmin test and vitamin test (vitamin A/vitamin B1/vitamin B2/vitamin B9/vitamin B12/vitamin C/vitamin D/vitamin E), and myocardial enzyme. MRI scanning of the whole brain, electroencephalography (EEG), electromyography (EMG), nerve conduction study (NCS), electrocardiograph (ECG) were conducted in all the patients with putative *SYNE1* pathogenic variants, except for that one patient (Patient II-2 of Family 2 in our study) did not receive EEG examination.

## Results

### Clinical Features

#### Family 1

##### Patient (III-1)

**Figure 1:**
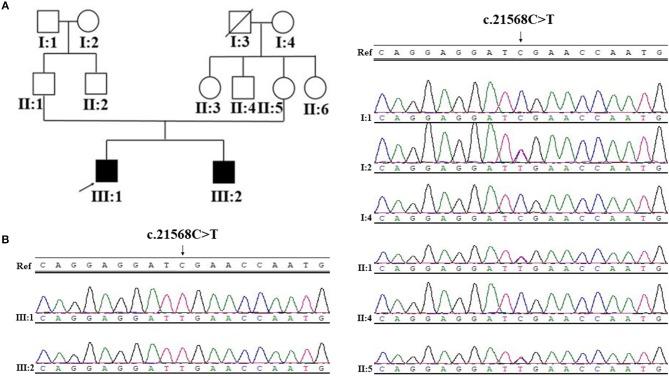
Pedigree structure, and Sanger sequencing of *SYNE1* variant (c.21568C>T) of Family 1. **(A)** Pedigree structure. **(B)** Segregation of *SYNE1* variant identified in subjects of Family 1, whose DNA sample were available. Ref: reference sequence in NCBI.

**Figure 2:**
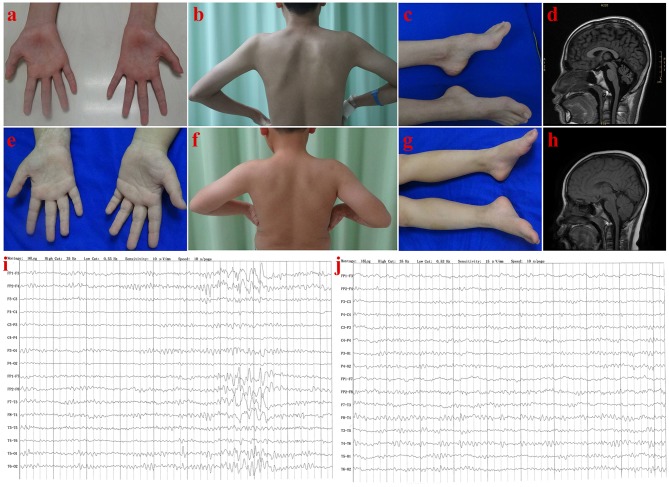
Clinical features of patients in Family 1. Patient III-1: Muscle atrophy in the thenar muscle, interosseous muscle of hands **(A)**, and shoulder girdle muscle **(B)**; bilateral pes cavus **(C)**; diffuse cerebellar atrophy on T1-weighted MRI **(D)**; EEG showed increased slow waves, paroxysmal sharp-slow wave in the frontal and temporal region **(I)**. Patient III-2: No muscle atrophy was found in hand **(E)**, and shoulder girdle muscle **(F)**, with normal appearance of feet **(G)**; T1-weighted MRI of the brain **(H)**, and EEG **(J)** were normal.

The proband (III-1) was a 16 year old boy from a non-consanguineous family with healthy parents (Figure 1A). He presented with weakness of upper and lower limbs, as well as hand muscle atrophy at the age 10. His medical history was insignificant. In the following time, muscle atrophy gradually spread to the forearm, upper arm, shoulder and pelvic girdles, thigh, crus, and foot muscles. At age 16, he showed an ataxic gait, upper limb ataxia, occasional diplopia, slurred speech, and cognitive decline. Neurological examinations revealed clinical signs of cerebellar ataxia, brisk tendon reflexes, and Babinski signs in lower limbs (Table 1). Muscle atrophy was mostly severe in the thenar muscles and interosseus muscles of hands (Figure 2A), and shoulder girdle muscle (Figure 2B). Bilateral pes cavus (Figure 2C) and mild ankle arthrogryposis were found. Serum CK level was 467 U/L (normal range, 50–310). MRI revealed diffuse cerebellar atrophy (Figure 2D). EEG showed increased slow waves, paroxysmal sharp-slow wave in the frontal and temporal region (Figure 2I). The motor nerve conduction velocities were normal and compound muscle action potentials amplitude decreased (Table 1). The sensory nerve conduction velocities and the sensory nerve action potentials were within the normal range (Table 1). F waves with increased amplitudes were found in the left tibial nerve (Supplementary Material_Figure S1).

**Table 1:** Clinical characteristics of *SYNE1* ataxia patients in this study.

**Clinical items**	**Patient (III-1) in Family 1**	**Patient (III-2) in Family 1**	**Patient (II-2) in Family2**
Gender	Male	Male	Male
Age at onset, y	10	9	15
Age at examination, y	16	9	22
Core clinical features	Ataxia+MND+Mental retardation+Arthrogryposis	Ataxia+MND+Low normal IQ	Ataxia+MND+Cognitive impairment
First symptom	MND	Ataxia	Cognitive impairment
MRI	Diffuse cerebellar atrophy	Normal	Diffuse cerebellar atrophy
EEG	Abnormal	Normal	Absent
EMG	Extensive neurogenic damage	Extensive neurogenic damage	Extensive neurogenic damage
MNCVs/CMAPs* (m/s, mV)			
Left median (E-W)	50.4/2.2	45.7/6.2	69.4/13.4
Right median (E-W)	NA**	44.2/5.4	61.6/15.2
Left ulnar (E-W)	NA**	46.8/2.9	63.7/13.9
Right ulnar (E-W)	52.2/8.4	47.1/2.0	65.3/16.1
Left peroneus (K-A)	44.9/4.6	47.4/3.2	55.6//11.8
Right peroneus (K-A)	43.5/8.1	49.6/2.7	53.7/7.3
Left tibial (PF-A)	36.8/7.6	46.8/9.3	52.8/21.2
Right tibial (PF-A)	36.2/5.2	50.4/9.7	54.9/24.2
SNCVs/SNAPs*** (m/s, μV)			
Left median (IIIF-W)	58.3/17.0	65.9/25.1	65.3/19.1
Right median (IIIF-W)	55.4/16.3	68.4/27.4	65.3/23.9
Left ulnar (VF-W)	50.0/9.4	59.3/14.5	68.8/14.6
Right ulnar (VF-W)	50.0/14.8	56.8/13.3	67.1/10.7
Left sural (A-sural)	47.4/18.4	53.2/21.3	45.5/11.1
Right sural (A-sural)	48.6/19.7	62.5/14.6	50.0/17.8
CK (U/L)	467.4	755.7	463.5
SARA	7	1.5	10
INAS	4	1	4
MMSE	29	28	24
MoCA	19	21	19
**WISC/WAIS**			
VIQ	72	88	82
PIQ	57	71	82
FIQ	62	78	81

A, Ankle; CK, Creatine Kinase; E, elbow; EEG, Electroencephalograph; EMG, electromyography; FIQ, Full Scale Intelligence Quotient; INAS, Inventory of Non-Ataxia Symptoms; K, Knee; MNCVs/CMAPs^*^, motor nerve conduction velocities (MNCVs) and compound muscle action potentials (CMAPs), the amplitudes of CMAPs from the distal stimulation site were recorded in this table; MMSE, Mini-Mental State Examination; MND, Motor Neuron Disorder; MoCA, Montreal Cognitive Assessment; MRI, Magnetic Resonance Imaging; NA^**^, Not available, due to the failure to elicit the waveform in the nerve conduction study; PF, popliteal fossa; PIQ, Performance Intelligence Quotient; SARA, Scale for Assessment and Rating of Ataxia; SNCVs/SNAPs^***^, sensory nerve conduction velocities (SNCVs) and sensory nerve action potentials (SNAPs), detected by the orthodromic method; W, wrist; WAIS, Wechsler Adult Intelligence Scale(age>16); WISC, Wechsler Intelligence Scale for Children (age ≤ 16); VIQ, Verbal Intelligence Quotient; IIIF, third finger; VF, fifth finger.

Needle EMG revealed very little or no spontaneous activity (fibrillation, positive sharp wave, and fasciculation potentials) in all investigated muscles (musculus quadratus labii inferioris, sternocleidomastoid, paravertebral muscle, biceps, abductor digiti minimi, tibialis anterior muscle). Large amplitude, long duration motor unit potentials, as well as reduced recruitment were shown in the muscles mentioned above. In summary, this patient (III-1) showed an obvious motor neuron disorder phenotype as its initial and dominant symptom, which mimicked juvenile-onset amyotrophic lateral sclerosis (ALS), accompanying with relatively slight cerebellar ataxia, mental retardation (FIQ < 70), and ankle arthrogryposis (Table 1).

##### Patient (III-2)

The younger brother (III-2) of the index patient was 9 years old. He reported no subjective symptoms at first. It was only when we told him the abnormalities in his routine neurological examination, he recalled that he showed difficulty in standing alone by one foot when playing games in the past 3 months. Physical examinations showed mild abnormalities in tandem step test, finger nose test of left hand, and normal muscle volume. EMG and NCV results were similar to his brother (III-1). The main clinical features were summarized in Table 1 and shown in Figure 2. In summary, this patient (III-2) showed mild ataxia and subclinical motor neuron disorder (summarized in Table 1).

#### Family 2

##### Patient (II-2)

**Figure 3:**
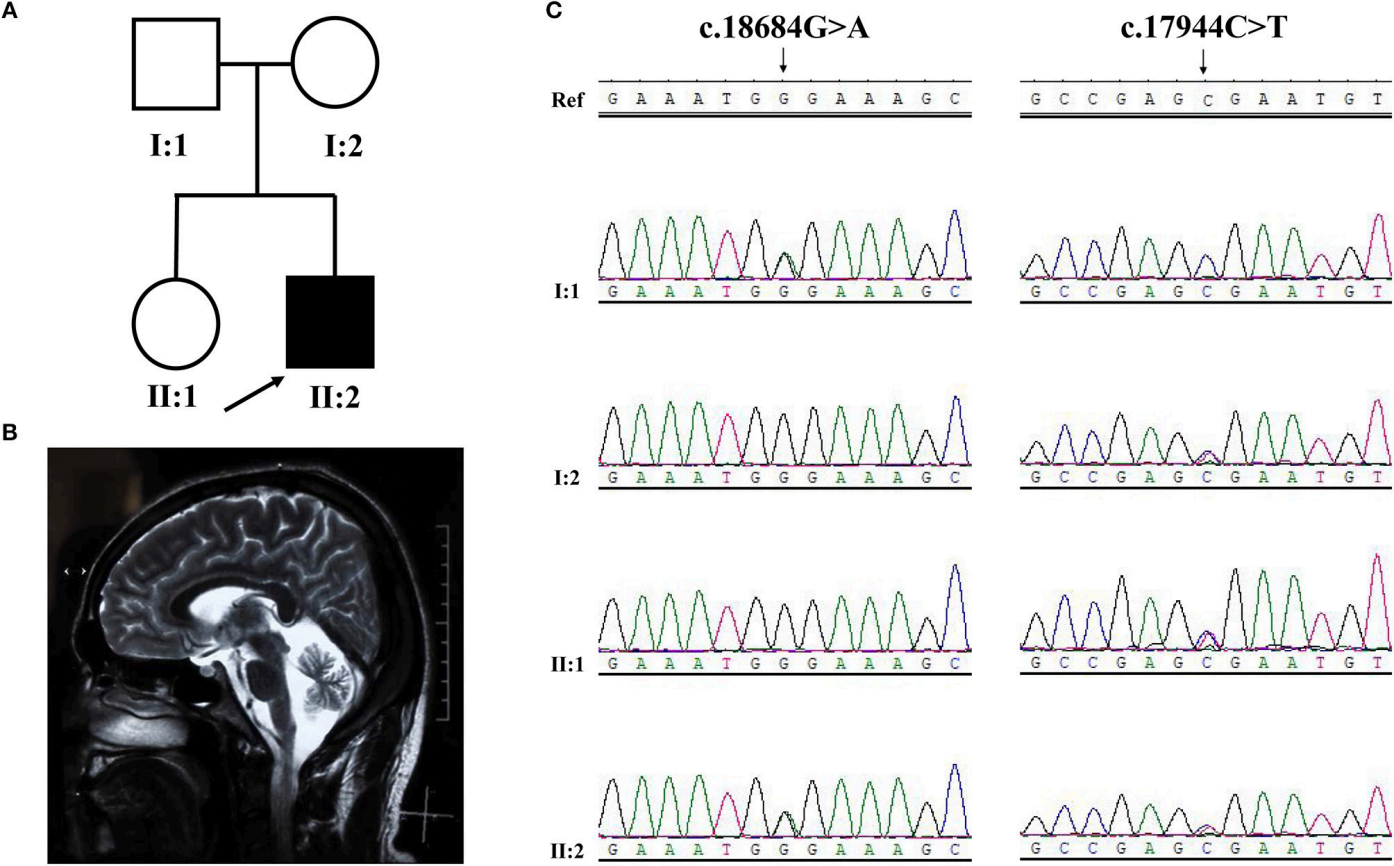
Pedigree structure, clinical features and Sanger sequencing of *SYNE1* variant of subjects in Family 2. **(A)** Pedigree structure. **(B)** Diffuse cerebellar atrophy on T2-weighted MRI. **(C)** Segregation of *SYNE1* variants identified in subjects of Family 2, whose DNA sample were available. Ref: reference sequence in NCBI.

The proband (II-2) was a 22 year old man from a non-consanguineous family with healthy parents and elder sister (Figure 3A). He firstly reported cognitive decline at the age 15, especially for the difficulty in study. At age 20, he developed a spastic-ataxic gait, followed by slurred speech. Neurological examinations revealed clinical signs of cerebellar ataxia, increased muscle tension in lower limbs, hyperreflexia in upper and lower limbs, positive pathologic reflexes, ankle clonus, with normal muscle strength, and volume (Table 1 and Figure 3). MRI of the brain showed diffuse cerebellar atrophy (Figure 3B). NCS demonstrated normal sensory nerve conduction and motor nerve conduction (Table 1). Needle EMG revealed very little or no spontaneous activity (fibrillation, positive sharp wave, and fasciculation potentials) in multiple muscles (musculus quadratus labii inferioris, sternocleidomastoid, paravertebral muscle, biceps, abductor digiti minimi, tibialis anterior muscle). Large amplitude, long duration motor unit potentials, as well as reduced recruitment were shown in these muscles. In summary, this patient (II-2) shows as a mild ataxia phenotype, and subclinical motor neuron disorder, with cognitive decline.

### Genetic Testing and Pathogenicity Assessment of *SYNE1* Variants

Among the 126 patients, 2 index patients were identified separately with the homozygous *SYNE1* variant (c.21568C>T, p.Arg7190Ter; Family 1) and compound heterozygous *SYNE1* variants (c.18684G>A, p.Trp6228Ter; c.17944C>T, p.Arg5982Ter; Family 2) in high-throughput screening.

#### Family 1

Sanger sequence identifies the segregation of the novel *SYNE1* truncating variant (c.21568C>T, p.Arg7190Ter) in family I (Figure 1B). This variant was absent or extremely rare in the population database (Table 2), and was not found in disease database (Clin Var, OMIM, HGMD, and literatures in PubMed till to Nov 6, 2018). It was predicted as disease causing by Mutation Taster (26), a high position-specific conservation score by GERP (30), and a likelihood of high pathogenicity by CADD (31) (Table 2). According to ACMG guidelines (29), the novel *SYNE1* mutation (c.21568C>T) is categorized to be the disease “pathogenic variant” because it belongs to PVS1 (non-sense mutation and loss of function of *SYNE1* has been validated to cause *SYNE1* ataxia), PM2 (extremely low population frequency if recessive), PP1 (segregation with phenotype in family), and PP4 (phenotype of this patient coincide with previously reported *SYNE1* related patients).

**Table 2:** Details of the two novel *SYNE1* variants found in this study.

**Position (GRCh37/hg19)**	**Exon (NM_033071.3)**	**cDNA change (NM_033071.3)**	**Protein change (NP_149062.1)**	**Mutation type**	**dbSNP**	**1000 genomes**	**ESP6500**	**ExAC**	**gnomAD**	**Mutation taster**	**GERP++ RS**	**CADD phred**	**ACMG category**
6:152542057-6:152542057	118/146	c.21568C>T	p.Arg7190Ter	Non-sense mutation	Novel	Novel	Novel	1.647e-5	4.064e-6	Disease causing	4.75	52	pathogenic
6:152583242-6:152583242	100/146	c.18684G>A	p.Trp6228Ter	Non-sense mutation	Novel	Novel	7.689e-5	8.236e-6	Novel	Disease causing	5.96	59	pathogenic

*Details of two novel SYNE1 variants identified in this study. Novel = Absent in the corresponding database. Mutation Taster, GERP++, and CADD are tools of in silico predictive algorithms recommended by ACMG guidelines (29)*.

#### Family 2

Sanger sequence identified the segregation of *SYNE1* variants (c.18684G>A; c.17944C>T) in Family 2 (Figure 3C). The variant (c.17944C>T; p.Arg5982Ter), has been reported to cause *SYNE1* ataxia previously (8). The novel truncating variant (c.18684G>A; p.Trp6228Ter) was extremely rare or absent in population database, absent in disease database (Clin Var, OMIM, HGMD, and literatures in PubMed), and predicted as disease causing by Mutation Taster, a high position-specific conservation score by GERP, and a likelihood of high pathogenicity by CADD (Table 2). Similar to above, the novel *SYNE1* variant (c.18684G>A) should also be categorized to be the disease “pathogenic variant” because it belongs to PVS1, PM2, PP1, PP4, as well as PM3 (for recessive disorders, detected in trans with a pathogenic variant) according to ACMG guidelines.

## Discussion

The recessive spinocerebellar ataxias (ARCAs or SCARs) are a complex group of neurodegenerative diseases with significant genetic and clinical heterogeneity, as well as regional and ethnic differences. Previous studies showed that *SYNE1* ataxia accounted for 5.3% (23/434), 6% (7/116), 2% (4/196), 0.9% (1/110) separately in four large independent cohorts of recessive and sporadic ataxia patients (7–10). In this study, we identified 2 unrelated index ataxia patients with novel *SYNE1* truncating variants, accounting for 1.6% (2/126) in this Chinese cohort of recessive and sporadic ataxia patients. This is the first report of *SYNE1* ataxia in China.

The recognition of phenotype of *SYNE1* ataxia has been going on for a long time. *SYNE1* ataxia was firstly described as an adult-onset, relatively pure cerebellar ataxia in 2007. In 2013, it was first reported as another form of early onset ataxia (age at onset: 6) with motor neuron disorder in Japan, which mimicked the phenotype of juvenile-onset amyotrophic lateral sclerosis (ALS) (2). Gradually, a growing number of ataxia patients with a wide range of extra-cerebellar neurological and non-neurological dysfunctions, have been found with pathogenic recessive *SYNE1* mutation, in different cohorts of ataxia patients from Europe (8–10, 14). Reported extra-cerebellar neurological and non-neurological dysfunctions included motor neuron disorder, mental retardation and cognitive impairment, depression, strabismus, ophthalmoparesis, bulging eyes, dystonia, reduced vibration sense, urge incontinence, kyphosis, scoliosis, pseudarthrosis clavicula, pes cavus, achilles tendon contractures, respiratory distress, macroglossia, sacral cyst, malrotation colon, splanchnectopia et al. Due to the above findings, Synofzik et al. proposed a new correct recognition of *SNYE1* ataxia as multisystemic ataxia in 2016 (10).

The present Chinese study provided new evidence supporting this suggestion. All the three ataxia patients shared a common character of ataxia syndrome, with motor neuron disorder, and mental retardation or cognitive decline. It is noteworthy that the patient III-1 in Family 1 showed a mild arthrogryposis in ankle, in addition to symptoms mentioned above. This is the second reported *SYNE1* ataxia case combined with arthrogryposis (information about the previously reported patient available in *Summary patient ID 52*, Supplementary Material_Table S3). EEG abnormalities in *SYNE1* ataxia had never been reported before. In this study, increased slow wave activity and paroxysmal sharp-slow waves in the frontal and temporal region were showed in the EEG of patient (III-1) in Family 1. The common pathologies contributing to EEG abnormalities, including seizure, cerebrovascular diseases, traumatic brain injury, central nervous system infections, central nervous demyelination, have been excluded by the clinical observation. Accordingly, the EEG abnormality in this patient, should be attributed to the cerebral cortex impairment caused by *SYNE1* mutation.

Another noteworthy finding in this study was EMG results, which may be beneficial for the differentiation diagnosis from juvenile ALS. Notwithstanding the different progression stage of these three patients, they shared the same characters in the Needle EMG: spontaneous activity (fibrillation, positive sharp wave, and fasciculation potentials) were very few or absent, but large amplitude, long duration motor unit potentials and reduced recruitment were very common. These results were different from typical EMG characters of juvenile ALS, which are obvious signs of denervation (positive sharp waves and fibrillation potentials) and fasciculation potentials, in combination with signs of reinnervation (large amplitude, long duration motor unit potentials, and reduced recruitment) in multiple regions (32). In contrast, EMG results in three patients in our study, revealed no signs of denervation, but only signs of reinnervation. These differences in EMG may be attributed to a slower pathological process of motor neurons in *SYNE1* ataxia, compared with juvenile ALS. Due to the similar clinical presentation, which includes both upper and lower motor neuron dysfunction, it is hard to make an accurate differential diagnosis between juvenile ALS and *SYNE1* ataxia, especially when the ataxia symptoms are very slight. There is a reason to assume that, the difference in EMG characters may add valuable information to the differential diagnosis between juvenile ALS and *SYNE1* ataxia. Research of EMG in more *SYNE1* ataxia patients is warranted to elucidate this hypothesis in the future.

The correlation between genotype and phenotype has not been clearly elucidated in *SYNE1* related diseases. Except for *SYNE1* ataxia, *SYNE1* variants have been found to cause other Mendelian disease, including Emery-Dreifuss muscular dystrophy 4 (EDMD4) (33), and arthrogryposis multiplex congenital (AMC) (34). In addition, it also has been associated with some other phenotype or complex diseases, including congenital muscular dystrophy (CMD), dilated cardiomyopathy (DCM), autism (AUT), mental retardation (MTD), schizophrenia.

**Figure 4:**
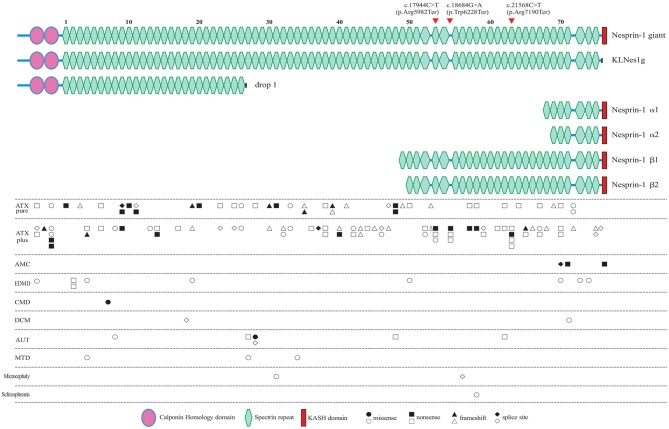
All of the reported pathogenic *SYNE1* variants, and their locations in the schematic diagram of Nesprin-1 and some of the major alternating isoforms of *SYNE1*. Locations of the variants found in the present study are denoted by the arrow head. Circle variants are missense variants, rectangles are non-sense variants, triangles are frameshift variants, and rhombus are splice site variants. Biallelic variants are shown in a solid color. ATX pure, patients with pure ataxia; ATX plus, ataxia patients with some other non-ataxia symptoms; AMC, arthrogryposis multiplex congenital; EDMD, Emery-Dreifuss muscular dystrophy; CMD, congenital muscular dystrophy; DCM, dilated cardiomyopathy; AUT, autism; MTD, mental retardation as the major syndrome.

*SYNE1* is one of the largest human gene consisting of 515, 716 bp, and 146 exons (35). *SYNE1* encodes a member of the spectrin family of structural proteins that participate in a complex that links the nucleoskeleton to the cytoskeleton (LINC), and links the plasma membrane to the actin cytoskeleton (36). *SYNE1* has multiple alternative start and termination sites, allowing the generation of alternating isoforms. The largest isoform (NM_182961) of *SYNE1* contains and encodes a 27748–base pair messenger RNA, and translates into an 8797–amino acid protein, noted as full-length Nesprin-1 (Nesprin-1 giant), started with two N-terminal actin-binding regions that comprise tandem paired calponin-homology domain (CH), followed by 74 spectrin repeats (SRs), and ending with the C-terminal klarsicht domain (KASH; Figure 4). Apart from full-length nesprin-1, at least 16 KASH- containing, 14 CH-containing, and 6 Spectrin repeat (SR)-only SYNE1 isoforms were reported (37). Razafsky et al. (38) noted that Nesprin-1 giant, and one of the CH-containing isoforms devoid of the KASH (KLNes1g) are specifically expressed in the central nervous system, and KLNes1g is mostly abundantly expressed in the cerebellum. One of KASH-containing isoforms (nesprin-1α2), devoid of CH and lots of SRs, is a predominantly muscle-specific isoform (39).

To explore the correlation between genotype and phenotype, we summarized all reported patients with *SYNE1* variant related phenotype (Figure 4, and Supplementary Material_Table S3).

In *SYNE1* ataxia patients, causative variants are distributed across the entire gene, except for the KASH domain, and without obvious hot mutation spot (Figure 4). This distribution of variant location supports the hypothesis that the loss of function of KLNes1g isoform, which is devoid of the KASH domain of Nesprin-1 giant, underlies the molecular etiology of ARCA1 (38). No obvious difference of variant distribution was observed between patients with pure ataxia (named as *ATX pure* in Figure 4), and patients with ataxia plus some other non-ataxia symptoms (named as *ATX plus* in Figure 4). Three interesting non-ataxia features including arthrogryposis, EEG abnormalities, and motor neuron disorder, were observed in our study which deserves further investigation. The patient (III-1) in Family 1 is the second reported *SYNE1* ataxia case with arthrogryposis. Coincidentally, the variants of the only two *SYNE1* ataxia patients with arthrogryposis, were all located in a very close region (SR61-SR63) of SYNE1 protein. In addition, the SR61-SR63 is very close to the KASH domain, a vital domain involved in the pathogenesis of AMC (34). This may suggest that variants in SR61-SR63 of SYNE1 protein would increase the susceptibility to arthrogryposis. It is possible that SR61-SR63 is an important overlapping functional region for both nerve and muscle tissue. As EEG abnormalities in *SYNE1* ataxia were only reported in our case [patient (III-1) in Family 1], it is still hard to evaluate the potential association between variant site and EEG abnormalities. The motor neuron disorder was the most frequent non-ataxia phenotype in *SYNE1* ataxia. However, no hot mutation spot was observed in all reported *SYNE1* ataxia patients with motor neuron disorders (Supplementary Material_Table S4). Nevertheless, more studies are needed to elucidate the potential correlation between variant sites and non-ataxia symptoms in *SYNE1* ataxia.

In AMC, all the causative mutations were located in KASH domain or neighboring region of KASH, which would be expected to affect most of the KASH-containing isoforms, especially for Nesprin-1α2, one of the predominantly muscle-specific isoforms of SYNE1 (39, 40). In EDMD, CMD, DCM, AUT, MTD, Microcephaly, and Schizophrenia, relationship between variant location and phenotype was unremarkable. The pathological mechanism underlying the *SYNE1* mutations responsible for phenotypic variations remains to be elucidated in future.

In conclusion, we reported the first two Chinese *SYNE1* ataxia families caused by novel *SYNE1* mutations, accompanied by motor neuron impairment, mental retardation, with or without arthrogryposis. Our findings expand the ethnic, phenotypic, and genetic diversity of *SYNE1* ataxia.

## Ethics Statement

This study was approved by the Ethics Committee of Xiangya Hospital, Central South University in China, which was in accordance with the ethical standards of the institutional and/or national research committee and with the 1964 Helsinki declaration and its later amendments or comparable ethical standards. And written informed consent was obtained from all subjects. We also obtained written and informed consent from the patients who gave specific permission to publish the data.

## Author Contributions

YP and HJ: conceived and designed the experiments. YP, WY, and ZC: performed the experiments. YP, ZC, HP, and PW: analyzed the data. YP, XuH, CW, XZ, XiH, TL, RQ, ZH, BT, and HJ: contributed reagents, materials, analysis tools. YP and ZC: contributed to the writing of the manuscript. ZH and HJ: reviewed and critiqued the first manuscript.

### Conflict of Interest Statement

The authors declare that the research was conducted in the absence of any commercial or financial relationships that could be construed as a potential conflict of interest.
