# Supplementary material for: Identifying SYNE1 Ataxia With Novel Mutations in a Chinese Population
Source: Front Neurol. 2018 Dec 20;9:1111. doi: 10.3389/fneur.2018.01111 (PMC6306413; doi:10.3389/fneur.2018.01111)
Supplement: Supplementary file 1 [file Data_Sheet_1.PDF]

Supplementary material

Identifying *SYNE1* ataxia with novel mutations in a Chinese population

Yun Peng[1], Wei Ye[1], Zhao Chen[1], Huirong Peng[1], Puzhi Wang[1], Xuan Hou[1], Chunrong Wang[1], Xin Zhou[1], Xiaocan Hou[1], Tianjiao Li[1], Rong Qiu[2], Zhengmao Hu[3], Beisha Tang[1,3,4,5,6,7,8] & Hong Jiang[1,3,5,9]

[1]Department of Neurology, Xiangya Hospital, Central South University, Changsha, Hunan, 410008, P. R. China

[2]School of Information Science and Engineering, Central South University, Changsha, Hunan, 410083, P. R. China

[3]Laboratory of Medical Genetics, Central South University, Changsha, Hunan, 410078, P. R. China

[4]National Clinical Research Center for Geriatric Diseases, Xiangya Hospital, Central South University, Changsha, Hunan 410008, P. R. China

[5]Key Laboratory of Hunan Province in Neurodegenerative Disorders, Central South University, Changsha, Hunan, 410008, P. R. China

[6]Parkinson's Disease Center of Beijing Institute for Brain Disorders, Beijing 100069, P. R. China

[7]Collaborative Innovation Center for Brain Science, Shanghai 200032, P. R. China

[8]Collaborative Innovation Center for Genetics and Development, Shanghai 200433, P. R. China

[9]Xinjiang Medical University, Xinjiang, 830011, P. R. China

Correspondence to: Dr. Hong Jiang, MD,

Department of Neurology, Xiangya Hospital,

Central South University,

Changsha 410008,

P. R. China

E-mail: jianghong73868@126.com

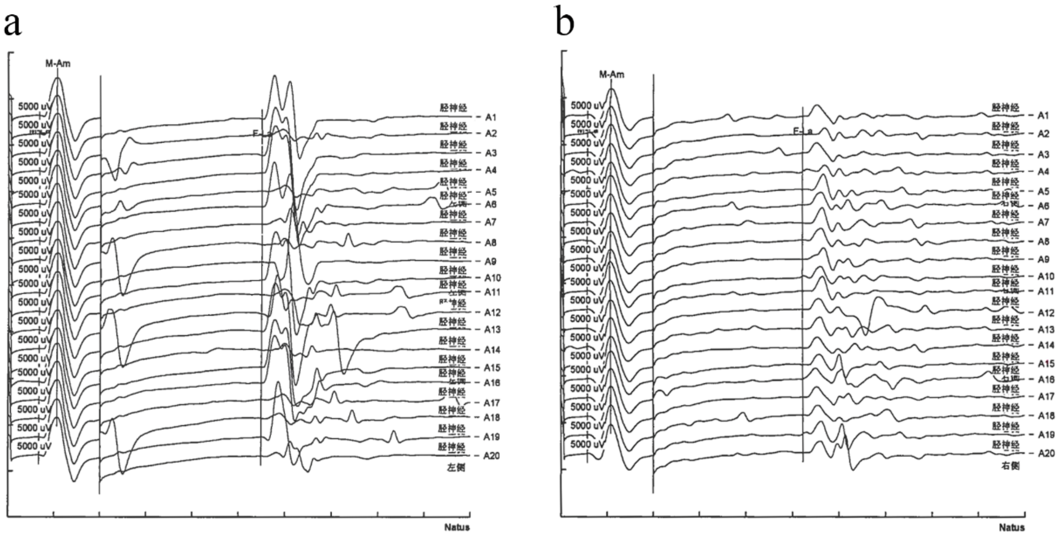

Supplementary material_Figure S1. F waves with the associated M waves in the left tibial nerve (a) and right tibial nerve (b) of Patient (III-1) in Family 1. The stimulation electrode was on the ankle, and the recording electrode was on the abductor hallucis. Twenty stimulations were performed on each side. The persistence of F wave was 100% on bilateral tibial nerve. Calibration per division in the vertical direction: 5 mV for M waves, 500μV for F-waves. Calibration per division in the horizontal direction: 10ms. According to the previous literatures, the mean F amplitude values are normally less than 5% of maximum M amplitude values. F waves with increased amplitude were shown in left tibial nerve (a) . The amplitudes of F waves in right tibial nerve were within the normal range (b).

Supplementary material _Table S1 Patients included in *SYNE1* mutation screening

**Pseudonym**	**Sex**	**Age at examination**	**Age at onset**	**Method of Genetic screening**	**Inheritance model**
S1	F	39	39	Whole-exome sequencing	AR
S2	F	15	13	Targeted panel sequencing	AR
S3	F	39	29	Targeted panel sequencing	S
S4	F	34	33	Targeted panel sequencing	S
S5	M	50	42	Targeted panel sequencing	S
S6	F	19	18	Targeted panel sequencing	S
S7	M	20	14	Targeted panel sequencing	S
S8	M	57	56	Targeted panel sequencing	S
S9	F	34	5	Targeted panel sequencing	AR
S10	F	45	30	Targeted panel sequencing	S
S11	M	27	17	Targeted panel sequencing	S
S12	M	38	30	Targeted panel sequencing	S
S13	M	31	11	Targeted panel sequencing	S
S14	M	44	44	Targeted panel sequencing	S
S15	M	25	16	Targeted panel sequencing	S
S16	M	34	25	Whole-exome sequencing	AR
S17	M	24	18	Targeted panel sequencing	S
S18	F	27	6	Whole-exome sequencing	AR
S19	M	59	54	Targeted panel sequencing	AR
S20	M	35	27	Whole-exome sequencing	AR
S21	M	45	27	Whole-exome sequencing	AR
S22	M	31	12	Whole-exome sequencing	AR
S23	F	14	9	Targeted panel sequencing	S
S24	M	31	30	Targeted panel sequencing	S
S25	M	35	28	Whole-exome sequencing	AR
S26	F	51	50	Targeted panel sequencing	S
S27	F	55	36	Whole-exome sequencing	AR
S28	M	57	54	Targeted panel sequencing	S
S29	M	24	16	Whole-exome sequencing	AR
S30	M	51	31	Targeted panel sequencing	AR
S31	F	37	29	Targeted panel sequencing	S

S32	M	51	46	Targeted panel sequencing	S
S33	M	51	47	Whole-exome sequencing	AR
S34	F	28	17	Targeted panel sequencing	S
S35	F	51	50	Targeted panel sequencing	S
S36	M	48	43	Targeted panel sequencing	S
S37	M	50	49	Targeted panel sequencing	S
S38	M	27	23	Whole-exome sequencing	AR
S39	M	23	22	Targeted panel sequencing	S
S40	F	52	50	Targeted panel sequencing	S
S41	F	48	46	Targeted panel sequencing	S
S42	M	49	49	Targeted panel sequencing	S
S43	M	25	20	Whole-exome sequencing	AR
S44	M	49	48	Whole-exome sequencing	S
S45	F	52	51	Targeted panel sequencing	S
S46	F	37	27	Targeted panel sequencing	S
S47	F	52	52	Targeted panel sequencing	S
S48	F	50	48	Targeted panel sequencing	S
S49	F	52	44	Targeted panel sequencing	S
S50	M	50	49	Targeted panel sequencing	S
S51	M	32	15	Targeted panel sequencing	AR
S52	M	32	15	Targeted panel sequencing	S
S53	M	36	34	Targeted panel sequencing	S
S54	F	17	14	Targeted panel sequencing	S
S55	F	60	58	Whole-exome sequencing	AR
S56	M	53	51	Targeted panel sequencing	S
S57	F	41	40	Targeted panel sequencing	S
S58	F	11	6	Targeted panel sequencing	S
S59	M	18	13	Whole-exome sequencing	S
S60	F	30	28	Targeted panel sequencing	S
S61	M	19	17	Whole-exome sequencing	AR
S62	F	23	9	Whole-exome sequencing	AR
S63	F	28	21	Targeted panel sequencing	S
S64	M	17	17	Targeted panel sequencing	S

S65	F	40	12	Targeted panel sequencing	S
S66	M	50	44	Whole-exome sequencing	AR
S67	M	38	33	Targeted panel sequencing	S
S68	M	36	29	Whole-exome sequencing	AR
S69	M	54	51	Whole-exome sequencing	S
S70	M	39	37	Whole-exome sequencing	AR
S71	F	31	27	Targeted panel sequencing	S
S72	F	54	53	Targeted panel sequencing	S
S73	F	47	46	Whole-exome sequencing	AR
S74	F	42	30	Whole-exome sequencing	AR
S75	M	33	24	Targeted panel sequencing	S
S76	M	19	16	Targeted panel sequencing	S
S77	M	47	37	Whole-exome sequencing	AR
S78	M	51	49	Targeted panel sequencing	S
S79	M	25	24	Targeted panel sequencing	S
S80	F	52	34	Targeted panel sequencing	S
S81	F	31	31	Targeted panel sequencing	S
S82	F	52	42	Targeted panel sequencing	S
S83	M	60	59	Targeted panel sequencing	AR
S84	M	18	14	Targeted panel sequencing	S
S85	F	15	10	Targeted panel sequencing	S
S86	F	30	29	Targeted panel sequencing	S
S87	M	15	13	Whole-exome sequencing	S
S88	M	12	10	Targeted panel sequencing	S
S89	M	21	12	Whole-exome sequencing	S
S90	M	24	23	Targeted panel sequencing	S
S91	M	24	23	Targeted panel sequencing	S
S92	F	49	47	Targeted panel sequencing	S
S93	F	49	48	Targeted panel sequencing	S
S94	F	40	37	Targeted panel sequencing	S
S95	F	40	40	Whole-exome sequencing	S
S96	F	48	48	Whole-exome sequencing	S
S97	F	26	22	Whole-exome sequencing	S

S98	M	34	33	Targeted panel sequencing	S
S99	F	52	50	Targeted panel sequencing	S
S100	M	22	14	Targeted panel sequencing	S
S101	F	29	15	Targeted panel sequencing	S
S102	M	26	21	Targeted panel sequencing	S
S103	F	36	31	Targeted panel sequencing	S
S104	M	59	33	Targeted panel sequencing	S
S105	F	51	11	Targeted panel sequencing	AR
S106	M	21	19	Targeted panel sequencing	S
S107	F	32	24	Whole-exome sequencing	S
S108	F	50	49	Targeted panel sequencing	S
S109	M	40	36	Targeted panel sequencing	S
S110	F	39	14	Targeted panel sequencing	AR
S111	M	43	33	Targeted panel sequencing	S
S112	F	43	33	Targeted panel sequencing	S
S113	F	51	39	Targeted panel sequencing	S
S114[a]	M	16	10	Whole-exome sequencing	AR
S115	M	16	13	Whole-exome sequencing	S
S116	F	59	58	Targeted panel sequencing	S
S117	F	31	25	Targeted panel sequencing	S
S118	F	53	51	Targeted panel sequencing	S
S119	F	58	57	Whole-exome sequencing	S
S120	F	55	51	Whole-exome sequencing	AR
S121[b]	M	22	15	Targeted panel sequencing	S
S122	M	47	46	Whole-exome sequencing	S
S123	M	51	50	Whole-exome sequencing	S
S124	F	24	19	Whole-exome sequencing	S
S125	F	20	17	Whole-exome sequencing	S
S126	F	46	42	Whole-exome sequencing	S

S=sporadic; AR=autosomal recessive; [a] indicates the proband in Family 1; [b] indicates the proband in Family 2.

Supplementary material_Table S2 Primers and PCR reaction conditions for Sanger sequencing of identified mutations in *SYNE1* gene in this study (NM_033071.3)

**Variant**	**Sequence (5'-3')**	**Amplicon size (bp)**	**Annealing temperature**
c.21568C>T	F: GTGGTAATCTGCTCATGAGTGTTG	431	55℃
	R: GAGACCTCTTAGTCTGATGCATGT		
c.18684G>A	F: ACACACACAAAAAGAGGGCAC	538	*touch down PCR* (65℃-59℃)
	R: CCAGGTCTGGTACACACAAT		
c.17944C>T	F: TCAGCCTCCCAAAGTGCTG	406	*touch down PCR* (65℃-59℃)
	R: CCACAGTCTTTCCTCCAGGC		

Supplementary material_Table S3 All of the reported *SYNE1* pathogenic variants and related phenotypes

Patient ID for summary	Case ID in original report	Ethnicity	Sex	AAO (years)	Clinical features	First sign	Exon/intron (NM_033071.3)	cDNA variant (NM_033071.3)	Protein change (NP_149062.1)	Protein change (NP_892006.3)	Domain in Nesprin-1 giant (NP_892006.3)	Variant type	Zygosity	Reference (PMID)
1[a]	NA/total 24	Fench-Canadian	NA	30.4 (17–46)	ATX	ATX	intron 81	c.15705-12A>G	Premature stop at position 5244	NA	NA	splice site	NA	17159980
1[a]	NA/total 24	Fench-Canadian	NA	30.4 (17–46)	ATX	ATX	intron 84	c.16177-2A>G	Premature stop at position 5402	NA	NA	splice site	NA	17159980
1[a]	NA/total 24	Fench-Canadian	NA	30.4 (17–46)	ATX	ATX	exon 93	c.17603_17607del	p.Asp5868AlafsTer13	p.Asp5939AlafsTer13	SR52-SR53	frameshift	NA	17159980
1[a]	NA/total 24	Fench-Canadian	NA	30.4 (17–46)	ATX	ATX	exon 126	c.22918C>T	p.Gln7640Ter	p.Gln7711Ter	SR67	nonsense	NA	17159980
1[a]	NA/total 24	Fench-Canadian	NA	30.4 (17–46)	ATX	ATX	exon 56	c.8716A>T	p.Arg2906Ter	p.Arg2899Ter	SR25	nonsense	NA	17159980
2	NA	Fench-Canadian	NA	NA	ATX	ATX	exon 72	c.11696_11697del	p.Met3899ArgfsTer7	p.Met3970ArgfsTer7	SR35	frameshift	c-het	17503513
2	NA	Fench-Canadian	NA	NA	ATX	ATX	exon 122	c.22156C>T	p.Gln7386Ter	p.Gln7386Ter	SR64	nonsense	c-het	17503513
3	NA	Fench-Canadian	F	30	ATX	ATX	intron 81	c.15705-12A>G	Premature stop at position 5244	NA	NA	splice site	NA	17503513
4	G11774	NA	M	early childhood	EDMD	EDMD	exon 139	c.25015G>T	p.Val8339Leu	p.Val8387Leu	SR72	missense	het	17761684
5	G12214	NA	M	52	EDMD	EDMD	exon 140	c.25237G>A	p.Glu8413Lys	p.Glu8461Lys	SR73	missense	het	17761684
6	G12552	NA	M	11	EDMD	EDMD	exon 133	c.24071G>A	p.Arg8024His	p.Arg8095His	SR70	missense	het	17761684
7	III-4	Palestinian	M	at birth	AMC+hypotonia	AMC	intron 133	c.24100-2A>G	NA	NA	SR70	splice site	hom	19542096
8	III-5	Palestinian	F	at birth	AMC+hypotonia	AMC	intron 133	c.24100-2A>G	NA	NA	SR70	splice site	hom	19542096
9	Patient	NA	NA	NA	DCM	DCM	exon 135	c.24422G>A	p.Arg8141His	p.Arg8212His	SR71	missense	het	19944109
10	patient 1	Japanese	F	6	ATX+MND	ATX	exon 124	c.22455dup	p.Ile7486AspfsTer3	p.Ile7557AspfsTer3	SR65	frameshift	hom	23325900
10	patient 1	Japanese	F	6	ATX+MND	ATX	exon 8	c.553G>C	p.Gly185Arg	p.Gly178Arg	CH2	missense	c-het, with two nonsense mutation	23325900
11	patient 2	Japanese	NA	36	ATX	ATX	exon 67	c.10789C>T	p.Arg3597Ter	p.Arg3590Ter	SR31	nonsense	hom	23325900
12	patient 3	Japanese	NA	27	ATX	ATX	exon 77	c.13601dup	p.Tyr4534Ter	p.Tyr4605Ter	SR41	frameshift	hom	23325900
13	AU-1600	NA	NA	NA	AUT+intellectual disability	AUT	exon 60	c.9637C>A	p.Leu3213Met	p.Leu3206Met	SR28	missense	hom	23352163
14	2-1369	NA	NA	NA	AUT	AUT	exon 27	c.3229C>T	p.Pro1077Ser	p.Pro1070Ser	SR8	missense	het	23849776
15	A-II-10	Fench-Canadian	M	30	ATX	ATX	exon 108	c.19859G>A	p.Trp6620Ter	p.Trp6691Ter	SR57	nonsense	c-het	23959263
15	A-II-10	Fench-Canadian	M	30	ATX	ATX	exon 7	c.373C>T	p.Arg125Ter	p.Arg118Ter	CH1	nonsense	c-het	23959263

16	A-II-8	Fench-Canadian	M	NA	ATX	ATX	exon 108	c.19859G>A	p.Trp6620Ter	p.Trp6691Ter	SR57	nonsense	c-het	23959263
16	A-II-8	Fench-Canadian	M	NA	ATX	ATX	exon 7	c.373C>T	p.Arg125Ter	p.Arg118Ter	CH1	nonsense	c-het	23959263
17	A-II-9	Fench-Canadian	F	29	ATX	ATX	exon 108	c.19859G>A	p.Trp6620Ter	p.Trp6691Ter	SR57	nonsense	c-het	23959263
17	A-II-9	Fench-Canadian	F	29	ATX	ATX	exon 7	c.373C>T	p.Arg125Ter	p.Arg118Ter	CH1	nonsense	c-het	23959263
18	B-III-1	Fench-Canadian	F	14	ATX	ATX	exon 116	c.21250C>T	p.Arg7084Ter	p.Arg7155Ter	SR62	nonsense	c-het	23959263
18	B-III-1	Fench-Canadian	F	14	ATX	ATX	exon 56	c.8716A>T	p.Arg2906Ter	p.Arg2899Ter	SR25	nonsense	c-het	23959263
19	B-III-2	Fench-Canadian	F	NA	ATX	ATX	exon 116	c.21250C>T	p.Arg7084Ter	p.Arg7155Ter	SR62	nonsense	c-het	23959263
19	B-III-2	Fench-Canadian	F	NA	ATX	ATX	exon 56	c.8716A>T	p.Arg2906Ter	p.Arg2899Ter	SR25	nonsense	c-het	23959263
20	sporadic A	Brazilian	NA	NA	ATX	NA	exon 31	c.3898C>T	p.Gln1300Ter	p.Gln1293Ter	SR10	nonsense	hom	23959263
21	sporadic B	French	NA	NA	ATX	NA	exon 64	c.10316_10320del	p.Ala3439ValfsTer4	p.Ala3432ValfsTer4	SR30	frameshift	hom	23959263
22	W10-1137	Sicilian	NA	NA	MTD	MTD	exon 71	c.11630T>C	p.Leu3877Ser	p.Leu3892Ser	SR34	missense	c-het	24123876
22	W10-1137	Sicilian	NA	NA	MTD	MTD	exon 19	c.1988A>G	p.Gln663Arg	p.Gln656Arg	SR4	missense	c-het	24123876
22	W10-1137	Sicilian	NA	NA	MTD	MTD	exon 58	c.9283G>A	p.Ala3095Thr	p.Ala3088Thr	SR27	missense	c-het	24123876
23	K168	NA	NA	NA	AMC	AMC	exon 135	c.24364C>T	p.Arg8122Ter	p.Arg8193Ter	SR71	nonsense	hom	24319099
24	Patient 2	NA	M	early childhood	EDMD+joint contractures	EDMD	exon 7	c.344A>G	p.Asn115Ser	p.Asn108Ser	CH1	missense	het	25091525
25	Patient 3	NA	M	3	EDMD+joint contractures+equinus foot deformity	EDMD	exon 7	c.344A>G	p.Asn115Ser	p.Asn108Ser	CH1	missense	het	25091525
26	ATX18	Spanish	F	NA	ATX	ATX	exon 109	c.20050C>T	p.Arg6684Ter	p.Arg6755Ter	SR58	nonsense	c-het	25133958
26	ATX18	Spanish	F	NA	ATX	ATX	exon 52	c.7938G>A	p.Trp2646Ter	p.Trp2639Ter	SR23	nonsense	c-het	25133958
27	ATX35	Palestinian	F	NA	ATX	ATX	intron 27	c.3417-10_3417delinsC	NA	NA	SR9	splice site	hom	25133958
28	ATX6	New Zealanders	M	NA	ATX	ATX	intron 34	c.4482+1G>T	NA	NA	SR12	splice site	c-het	25133958
28	ATX6	New Zealanders	M	NA	ATX	ATX	exon 60	c.9646A>T	p.Lys3216Ter	p.Lys3209Ter	SR28	nonsense	c-het	25133958
29	ATX63	European	M	NA	ATX	ATX	exon 69	c.11181G>T	p.Met3727Ile	p.Met3727Ile	SR33	missense	c-het	25133958
29	ATX63	European	M	NA	ATX	ATX	exon 136	c.24617G>C	p.Ser8206Thr	p.Ser8277Thr	SR71-SR72	missense	c-het	25133958
30	ATX69	European	M	NA	ATX+polyneuropathy	NA	exon 90	c.17016T>G	p.Tyr5672Ter	p.Tyr5743Ter	SR52	nonsense	c-het	25133958
30	ATX69	European	M	NA	ATX+polyneuropathy	NA	exon 27	c.3209T>C	p.Val1070Ala	p.Val1063Ala	SR8	missense	c-het	25133958
31	12JC-p	NA	NA	23	schizophrenia	schizophrenia	exon 108	c.19898A>T	p.Gln6633Leu	p.Gln6704Leu	SR58	missense	het	25420024
32	SYNE#1-1	Turkish	M	20	ATX+MND	NA	NA	NA	p.Gln7573Ter	NA	NA	NA	hom	25681989
33	SYNE#1-2	Turkish	M	21	ATX+MND	NA	NA	NA	p.Gln7573Ter	NA	NA	NA	hom	25681989
34	SYNE#2-1	Turkish	F	26	ATX+MND	NA	NA	NA	p.Gln7771Ter	NA	NA	NA	hom	25681989

35	SYNE#2-2	Turkish	F	17	ATX+MND	NA	NA	NA	p.Gln7771Ter	NA	NA	NA	hom	25681989
36	SYNE#2-3	Turkish	F	30	ATX+MND	NA	NA	NA	p.Gln7771Ter	NA	NA	NA	hom	25681989
37	4	English	F	40	ATX	ATX	exon 18	c.1784del	p.Asn595MetfsTer12	p.Asn588MetfsTer12	SR3	frameshift	c-het	25976027
37	4	English	F	40	ATX	ATX	exon 58	c.9169C>G	p.Leu3057Val	p. Leu3050Val	SR26	missense	c-het	25976027
38	SCAR8 patient	Algerian	NA	7	ATX	ATX	exon 30	c.3736G>T	p.Glu1246Ter	p.Glu1239Ter	SR9	nonsense	hom	26068213
39	NA	NA	NA	NA	AUT	NA	exon 83	c.15898C>T	p.Arg5300Ter	p.Arg5371Ter	SR48	nonsense	het	26185613
40	NA	NA	NA	NA	AUT	NA	exon 116	c.21250C>T	p.Arg7084Ter	p.Arg7155Ter	SR62	nonsense	het	26185613
41	NA	NA	NA	NA	AUT	NA	exon 59	c.9370G>T	p.Gly3124Ter	p.Gly3117Ter	SR27	nonsense	het	26185613
42	NA	NA	NA	NA	AUT	NA	intron 61	c.9828+1G>T	NA	NA	SR28	splice site	het	26185613
43	BAB5723	NA	F	NA	microcephaly, hypoplasia of the brain stem and cerebellum, delayed myelination	NA	exon 67	c.10748G>A	p.Arg3583Gln	p.Arg3576Gln	SR31	missense	het	26539891
43	BAB5723	NA	F	NA	microcephaly, hypoplasia of the brain stem and cerebellum, delayed myelination	NA	intron 105	c.19479+3G>A	NA	NA	SR56	splice site	het	26539891
44	30	NA	F	at birth	EDMD4	EDMD4	exon 15	c.1399A>T	p.Lys467Ter	p.Lys460Ter	SR2	nonsense	het	27066551
45	54	NA	M	53	EDMD4	EDMD4	exon 16	c.1507T>A	p.Ser503Thr	p.Ser496Thr	SR2	missense	het	27066551
46	7	NA	M	at birth	EDMD4	EDMD4	exon 86	c.16388A>C	p.Glu5463Ala	p.Glu5534Ala	SR50	missense	het	27066551
47	7	NA	M	at birth	EDMD4	EDMD4	exon 18	c.1859C>T	p.Ser620Phe	p.Ser613Phe	SR4	missense	het	27066551
48	10-1	German	F	21	ATX+MND+urge incontinence	NA	intron 107	c.19855-1G>C	NA	NA	SR57	splice site	hom	27086870
49	1-1	Turkish	M	22	ATX	ATX	exon77	c.13086delC	p.His4362GlnfsTer2	p.His4433GlnfsTer2	SR39	frameshift	hom	27086870
50	11-1	Turkish	F	18	ATX+MND+depression	NA	exon 108	c.19897C>T	p.Gln6633Ter	p.Gln6704Ter	SR58	nonsense	hom	27086870
51	12-1	Belgian	F	6	ATX+MND+MTD +respiratory distress+pes vacus+strabism+Respiratory distress+sacral cyst+malrotation colon+pseudarthrosis clavicula+2 kidneys right sided, kyphosis+scoliosis+CK elevation+cataract+ myoclonus	NA	exon 114	c.20935C>T	p.Arg6979Ter	p.Arg7050Ter	SR61	nonsense	c-het	27086870

51	12-1	Belgian	F	6	ATX+MND+MTD +respiratory distress+pes vacus+strabism+Respiratory distress+sacral cyst+malrotation colon+pseudarthrosis clavicula+2 kidneys right sided, kyphosis+scoliosis+CK elevation+cataract+myoclonus	NA	exon 118	c.21528C>A	p.Tyr7176Ter	p.Tyr7247Ter	SR63	nonsense	c-het	27086870
52	12-2	Belgian	F	6	ATX+MND+MTD +respiratory distress+hypertelorism+CK elevation+pes cavus+ hyperlaxity+ achilles tendon contractures	NA	exon 114	c.20935C>T	p.Arg6979Ter	p.Arg7050Ter	SR61	nonsense	c-het	27086870
52	12-2	Belgian	M	6	ATX+MND+MTD +respiratory distress+hypertelorism+CK elevation+pes cavus+ hyperlaxity+ achilles tendon contractures	NA	exon 118	c.21528C>A	p.Tyr7176Ter	p.Tyr7247Ter	SR63	nonsense	c-het	27086870
53	13-1	Moroccan	F	30	ATX+MND	NA	exon 9	c.682C>T	p.Arg228Ter	p.Arg221Ter	CH2	nonsense	hom	27086870
54	13-2	Moroccan	F	28	ATX+MND	NA	exon 9	c.682C>T	p.Arg228Ter	p.Arg221Ter	CH2	nonsense	hom	27086870
55	14-1	German	M	15	ATX+MND+pes cavus	NA	exon 93	c.17480dup	p.Tyr5827Ter	p.Tyr5898Ter	SR52-SR53	frameshift	c-het	27086870
55	14-1	German	M	15	ATX+MND+pes cavus	NA	exon 111	c.20380C>T	p.Gln6794Ter	p.Gln6865Ter	SR59	nonsense	c-het	27086870
56	15-1	French	M	27	ATX+ophtalmoparesis+mild microretrognathism +unilateral ptosis	NA	intron 75	c.12315+1G>A	NA	NA	SR37	splice site	hom	27086870
57	15-2	French	F	17	ATX+scoliosis+seizures	NA	intron 75	c.12315+1G>A	NA	NA	SR37	splice site	hom	27086870
58	16-1	French	F	27	ATX	ATX	exon 47	c.6978G>A	p.Trp2326Ter	p.Trp2319Ter	SR20	nonsense	hom	27086870
59	17-1	French	F	7	ATX+pes cavus	NA	exon 30	c.3736G>T	p.Glu1246Ter	p.Glu1239Ter	SR9	nonsense	hom	27086870

60	18-1	German	F	19	ATX	ATX	exon 85	c.16294_16300del	p.Lys5432LeufsTer25	p.Lys5503LeufsTer25	SR49	frameshift	c-het	27086870
60	18-1	German	F	19	ATX	ATX	exon 133	c.24054G>A	p.Trp8018Ter	p.Trp8089Ter	SR70	nonsense	c-het	27086870
61	19-1	Italian	F	10	ATX+MND+ophtalmoparesis+Slow saccade	NA	exon 95	c.17944C>T	p.Arg5982Ter	p.Arg6053Ter	SR53	nonsense	hom	27086870
62	20-1	French	F	24	ATX+MND+reduced vibration sense+urge incontinence+bulging eyes+upper limb dystonia	NA	exon 24	c.2776A>T	p.Lys926Ter	p.Lys919Ter	SR6	nonsense	c-het	27086870
62	20-1	French	F	24	ATX+MND+reduced vibration sense+urge incontinence+bulging eyes+upper limb dystonia	NA	exon 9	c.659T>C	p.Phe220Ser	p.Phe213Ser	CH2	missense	c-het	27086870
63	2-1	German	M	24	ATX+slow saccades	NA	exon 77	c.14569C>T	p.Gln4857Ter	p.Gln4928Ter	SR44	nonsense	c-het	27086870
63	2-1	German	M	24	ATX+slow saccades	NA	intron 5	c.309+1G>A	NA	NA	CH1	splice site	c-het	27086870
64	21-1	Italian	F	16	ATX+MND+urge incontinence	NA	exon 77	c.14261dup	p.Gln4755ProfsTer15	p.Gln4826ProfsTer15	SR43	frameshift	c-het	27086870
64	21-1	Italian	F	16	ATX+MND+urge incontinence	NA	exon 41	c.6034C>T	p.Arg2012Ter	p.Arg2005Ter	SR17	nonsense	c-het	27086870
65	22-1	Italian	M	20	ATX+MND+scoliosis	NA	exon 9	c.727C>T	p.Arg243Ter	p.Arg236Ter	CH2	nonsense	hom	27086870
66	23-1	Italian	M	10	ATX+MND+mental retardation+ slow saccades+restrictive ventilatory defect+pes cavus	NA	intron 109	c.20183+1G>C	NA	NA	SR59	splice site	c-het	27086870
66	23-1	Italian	M	10	ATX+MND+MTD + slow saccades+restrictive ventilatory defect+pes cavus	NA	exon 132	c.23782C>T	:p.Arg7928Ter	p.Arg7999Ter	SR70	nonsense	c-het	27086870
67	3-1	Turkish	F	25	ATX	ATX	exon 82	c.15760C>T	p.Arg5254Ter	p.Arg5325Ter	SR48	nonsense	hom	27086870
68	4-1	German	F	40	ATX+urge incontinence	NA	exon 81	c.15665_15666del	p.Leu5222HisfsTer21	p.Leu5293HisfsTer21	SR47	frameshift	c-het	27086870
68	4-1	German	F	40	ATX+urge incontinence	NA	exon 133	c.24025C>T	p.Gln8009Ter	p.Gln8080Ter	SR70	nonsense	c-het	27086870
69	5-1	Italian	M	35	ATX+MND	NA	exon 132	c.23782C>T	p.Arg7928Ter	p.Arg7999Ter	SR70	nonsense	c-het	27086870
69	5-1	Italian	M	35	ATX+MND	NA	exon 55	c.8627_8628insAT	p.Met2876IlefsTer19	p.Met2869IlefsTer19	SR25	frameshift	c-het	27086870
70	6-1	Italian	F	20	ATX+scoliosis	NA	exon 77	c.14255del	p.Gly4752GlufsTer10	p.Gly4823GlufsTer10	SR43	frameshift	c-het	27086870

70	6-1	Italian	F	20	ATX+scoliosis	NA	exon 7	c.395T>A	p.Leu132Ter	p.Leu125Ter	CH1	nonsense	c-het	27086870
71	7-1	Italian	F	36	ATX	ATX	exon 80	c.15419A>G	p.Asp5140Glyfs *1	p.Asp5211Glyfs *1	SR47	splice site(crypti c)	c-het	27086870
71	7-1	Italian	F	36	ATX	ATX	exon 131	c.23684_23685in sACGCCTGTGC CACTGATGCC GAGTG	p.Cys7895Ter	p.Cys7966Ter	SR69	frameshift	c-het	27086870
72	8-1	Italian	F	25	ATX+scoliosis+kyp hosis	NA	intron 14	c.25516-1G>A	NA	NA	SR74	splice site	c-het	27086870
72	8-1	Italian	F	25	ATX+scoliosis+kyp hosis	NA	intron 52	c.8026-2A>G	NA	NA	SR23	splice site	c-het	27086870
73	9-1	German	M	28	ATX+MND+mild macroglossia+esotro pia	NA	exon 73	c.11908C>T	p.Arg3970Ter	p.Arg4041Ter	SR36	nonsense	c-het	27086870
73	9-1	German	M	28	ATX+MND+mild macroglossia+esotro pia	NA	exon 117	c.21316C>T	p.Gln7106Ter	p.Gln7177Ter	SR62	nonsense	c-het	27086870
74	I-I:1	English	M	40	ATX+cognitive impairment	ATX	exon 98	c.18431G>A	p.Trp6144Ter	p.Trp6215Ter	SR54-SR55	nonsense	c-het	27178001
74	I-I:1	English	M	40	ATX+cognitive impairment	ATX	exon 18	c.1849G>T	:p.Glu617Ter	p.Glu610Ter	SR4	nonsense	c-het	27178001
75	I-I:4	English	F	32	ATX+cognitive impairment	ATX	exon 98	c.18431G>A	p.Trp6144Ter	p.Trp6215Ter	SR54-SR55	nonsense	c-het	27178001
75	I-I:4	English	F	32	ATX+cognitive impairment	ATX	exon 18	c.1849G>T	:p.Glu617Ter	p.Glu610Ter	SR4	nonsense	c-het	27178001
76	II-II:1	Turkish	F	18	ATX+cognitive impairment	ATX	exon 108	c.19897C>T	p.Gln6633Ter	p.Gln6633Ter	SR57	nonsense	hom	27178001
77	III-III:1	SriLankan	M	22	ATX+MND+cognit ive impairment	ATX	exon 77	c.13429C>T	p.Gln4477Ter	p.Gln4548Ter	SR40	nonsense	hom	27178001
78	1-1	Turkish	M	17	ATX+MND	NA	exon 98	c.18370C>T	p.Gln6124Ter	p.Gln6195Ter	SR54-SR55	nonsense	hom	27197992
79	1-2	Turkish	M	11	ATX+MND+pes equinovarus	NA	exon 98	c.18370C>T	p.Gln6124Ter	p.Gln6195Ter	SR54-SR55	nonsense	hom	27197992
80	2-1	Belgian	F	6	ATX+MND+restric tive lung function	NA	exon 129	c.23341_23342de l	p.Ala7781IlefsTe r2	p.Ala7852IlefsTe r2	SR68	frameshift	c-het	27197992
80	2-1	Belgian	F	6	ATX+MND+restric tive lung function	NA	exon 36	c.4753C>T	p.Pro1585Ser	p.Pro1578Ser	SR13	missense	c-het	27197992
81	3-1	Turkish	F	28	ATX+MND	NA	exon 77	c.13354_13357de l	p.Glu4452SerfsT er34	p.Glu4523SerfsT er34	SR40	frameshift	c-het	27197992
81	3-1	Turkish	F	28	ATX+MND	NA	exon 136	c.24601C>T	p.Arg8201Ter	p.Arg8272Ter	SR71-SR72	frameshift	c-het	27197992
82	4-1	German	F	42	ATX+cognitive impairment	NA	exon 78	c.14816_14819de l	p.Glu4939Valfs Ter13	p.Glu5010Valfs Ter13	SR45	frameshift	c-het	27197992
82	4-1	German	F	42	ATX+cognitive impairment	NA	intron 79	c.15225+2T>A	NA	NA	SR46	splice site	c-het	27197992

83	5-1	German	M	23	ATX+MND+writer's cramp+polyneuropathy	NA	exon 65	c.10400_10401del	p.Tyr3467CysfsTer28	p.Tyr3460CysfsTer28	SR30	frameshift	c-het	27197992
83	5-1	German	M	23	ATX+MND+writer's cramp+polyneuropathy	NA	exon 68	c.10951_10958del	p.Leu3651GlufsTer51	p.Leu3666GlufsTer51	SR32	frameshift	c-het	27197992
84	6-1	Italian	M	7	ATX+MND+cognitive impairment+depression	NA	exon 18	c.1924dup	p.Met642AsnfsTer35	p.Met635AsnfsTer35	SR4	frameshift	hom	27197992
85	7-1	Italian	M	6	ATX+MND+hammer-toes+motor polyneuropathy	NA	exon 119	c.21758C>A	p.Ser7253Ter	p.Ser7324Ter	SR63	nonsense	c-het	27197992
85	7-1	Italian	M	6	ATX+MND+hammer-toes+motor polyneuropathy	NA	exon 141	c.25511del	p.Cys8504SerfsTer7	p.Cys8552SerfsTer7	SR74	frameshift	c-het	27197992
86	patient 1	Turkish	M	at birth	AMC+congenital muscular weakness	AMC+congenital muscular weakness	exon 146	c.26092C>T	p.Arg8698Ter	p.Arg8746Ter	KASH	nonsense	hom	27782104
87	case1	Saudi	M	24	ATX+white matter abnormalities mimicking multiple sclerosis	ATX	exon 77	c.13878G>T	p.Met4626Ile	p.Met4697lle	SR42	missense	c-het	28017257
87	case1	Saudi	M	24	ATX+white matter abnormalities mimicking multiple sclerosis	ATX	exon 91	c.17270C>G	p.Thr5757Arg	p.Thr5828Arg	SR52	missense	c-het	28017257
88	case2	Saudi	F	22	ATX+white matter abnormalities mimicking multiple sclerosis	ATX	exon 77	c.13878G>T	p.Met4626Ile	p.Met4697lle	SR42	missense	c-het	28017257
88	case2	Saudi	F	22	ATX+white matter abnormalities mimicking multiple sclerosis	ATX	exon 91	c.17270C>G	p.Thr5757Arg	p.Thr5828Arg	SR52	missense	c-het	28017257
89	III: 6	Chinese	M	12	EDMD	EDMD	exon 47	c.6910G>A	p.Gly2304Arg	p.Gly2297Arg	SR19	missense	het	28583108
90	fater of NCG_00024	NA	M	NA	DCM	DCM	inron 43	c.6403-1G>A	NA	NA	SR18	splice site	het	28611029
91	NCG_00024	NA	NA	NA	DCM	DCM	intron 43	c.6403-1G>A	NA	NA	SR18	splice site	het	28611029

92	AT12-1	Turkish	F	26	ATX+MND+systemic lupus erythematosus with arthritis+urinary incontinence+migrainous type	ATX	exon 77	c.13086del	p.His4362GlnfsTer2	p.His4433GlnfsTer2	SR39	frameshift	hom	28687974
93	AT12-2	Turkish	M	23	ATX	ATX	exon 77	c.13086del	p.His4362GlnfsTer2	p.His4433GlnfsTer2	SR39	frameshift	hom	28687974
94	AT12-3	Turkish	F	26	ATX+depression+type 1 diabetes+hyperlipidemia+asymptomatic mitral valve prolapse	ATX	exon 77	c.13086del	p.His4362GlnfsTer2	p.His4433GlnfsTer2	SR39	frameshift	hom	28687974
95	AT12-4	Turkish	M	21	ATX	ATX	exon 77	c.13086del	p.His4362GlnfsTer2	p.His4433GlnfsTer2	SR39	frameshift	hom	28687974
96	case 1	Chinese	F	NB	CMD+mildly enlarged heart	CMD	exon 24	c.2902C>T	p.Arg968Trp	p.Arg961Trp	SR7	missense	hom	28818390
97	patient1	Japanese	F	22	ATX	ATX	exon 46	c.6843del	p.Gln2282SerfsTer3	p.Gln2275SerfsTer3	SR19	frameshift	hom	29081981
98	patient2	Japanese	M	30	ATX	ATX	exon 46	c.6843del	p.Gln2282SerfsTer3	p.Gln2275SerfsTer3	SR19	frameshift	hom	29081981
99	patient3	Japanese	F	30	ATX	ATX	exon 46	c.6843del	p.Gln2282SerfsTer3	p.Gln2275SerfsTer3	SR19	frameshift	hom	29081981
100	AAR-163-6	NA	NA	NA	ATX	ATX	exon 83	c.16015C>T	p.Arg5339Ter	p.Arg5410Ter	SR48	nonsense	hom	29482223
101	AAR-241-13	NA	NA	NA	ATX	ATX	exon 136	c.24718C>G	p.Gln8240Glu	p.Gln8311Glu	SR71-SR72	missense	c-het(3)	29482223
101	AAR-241-13	NA	NA	NA	ATX	ATX	exon 24	c.2776A>T	p.Lys926Ter	p.Lys919Ter	SR6	nonsense	c-het(3)	29482223
101	AAR-241-13	NA	NA	NA	ATX	ATX	exon 9	c.659T>C	p.Phe220Ser	p.Phe213Ser	CH2	missense	c-het(3)	29482223
102	AAR-536-4	NA	NA	NA	ATX	ATX	exon 35	c.4582C>T	p.Arg1528Ter	p.Arg1521Ter	SR12	nonsense	hom	29482223
103	AAR-61-11	NA	NA	NA	ATX	ATX	exon 72	c.11733_11734insTT	p.Leu3912PhefsTer4	p.Leu3983PhefsTer4	SR35	frameshift	hom	29482223
104	SAL- 399-1023	NA	NA	NA	ATX+sensory impairment	NA	exon 69	c.11142G>T	p.Lys3714Asn	p.Lys3729Asn	SR33	missense	c-het	29482223
104	SAL- 399-1023	NA	NA	NA	ATX+sensory impairment	NA	exon 127	c.22966G>A	p.Asp7656Asn	p.Asp7727Asn	SR67	missense	c-het	29482223
105	SAL- 399-573	NA	NA	NA	ATX+spastic syndrome	NA	exon 77	c.14060T>G	p.Leu4687Ter	p.Leu4758Ter	SR42	nonsense	c-het	29482223
105	SAL- 399-573	NA	NA	NA	ATX+spastic syndrome	NA	exon 128	c.23133G>A	p.Trp7711Ter	p.Trp7782Ter	SR67	nonsense	c-het	29482223
106	Patient 1	Brazilian	F	16	ATX+MND	ATX	exon 119	c.21676A>T	p.Lys7226Ter	p.Lys7297Ter	SR63	nonsense	NA	29801895
107	Patient 2	Brazilian	M	36	ATX	ATX	exon 76	c.12567G>A	p.Trp4189Ter	p.Trp4260Ter	SR38	nonsense	NA	29801895
108	Patient 3	Brazilian	M	23	ATX	ATX	exon 86	c.16454G>A	p.Trp5485Ter	p.Trp5556Ter	SR50	nonsense	NA	29801895
109	Patient 4	Brazilian	M	24	ATX	ATX	exon 77	c. 12808-12809delGT	p.Thr4270Ter	p.Thr4341Ter	SR39	frameshift	NA	29801895

110	Patient 5	Brazilian	F	37	ATX+dystonia	dystonia	exon 76	c.12567G>A	p.Trp4189Ter	p.Trp4260Ter	SR38	nonsense	NA	29801895
111	Patient 6	Brazilian	F	42	ATX+dystonia	ATX	exon 76	c.12567G>A	p.Trp4189Ter	p.Trp4260Ter	SR38	nonsense	NA	29801895
112	Patient 074	North American	M	20	ATX	ATX	exon 12	c.1042G>T	p.Glu348Ter	p.Glu341Ter	SR1	nonsense	hom	29915382
113	Patient 076	North American	F	25	ATX+ Spasticity	NA	exon 122	c.22195G>T	p.Glu7399Ter	p.Glu7470Ter	SR65	nonsense	c-het	29915382
113	Patient 076	North American	F	25	ATX+ Spasticity	NA	exon 125	c.22788dup	p.Leu7597Thrfs*12	p.Leu7668Thrfs*12	SR66	frameshift	c-het	29915382
114	Patient 078	North American	F	10	ATX+ developmental delay+ lower extremity sensory loss and weakness	NA	exon 47	c.6898del	p.Glu2300Lysfs*2	p.Glu2293Lysfs*2	SR19	frameshift	c-het	29915382
114	Patient 078	North American	F	10	ATX+ developmental delay+ lower extremity sensory loss and weakness	NA	intron 83	c.16024-13C>G	NA	NA	NA	NA	c-het	29915382
115	Patient 080	North American	M	34	ATX+ facial masking	NA	exon 8	c.503_504del	p.Ser168Ter	p.Ser161Ter	CH1-CH2	frameshift	hom	29915382
116	Patient 081	North American	F	30	ATX+ Mild hyperreflexia	NA	exon 39	c.5182G>T	p.Glu1728Ter	p.Glu1721Ter	SR14	nonsense	hom	29915382
117	Monozygous twins	Caucasian	F	0.416	ATX+cerebellar hypoplasia+cognitive impairment	Hypotonia+ developmental delay	exon 67	c.10805A>G	p.Asn3602Ser	p.Asn3595Ser	SR32	missense	c-het	30275942
117	Monozygous twins	Caucasian	F	0.416	ATX+cerebellar hypoplasia+cognitive impairment	Hypotonia+ developmental delay	exon 95	c.17878G>A	p.Glu5960Lys	p.Glu6031Lys	SR53	missense	c-het	30275942
118	Case 1	Korean	M	39	ATX+autonomic dysfunction+cognitive impairment	ATX	exon 39	c.5242C>T	p.Gln1748Ter	p.Gln1741Ter	SR14	nonsense	c-het	30119932
118	Case 1	Korean	M	39	ATX+autonomic dysfunction+cognitive impairment	ATX	intron 145	c.26009+1G>A	NA	NA	SR74-KASH	splice site	c-het	30119932
119	Case 2	Korean	M	39	ATX+ autonomic dysfunction+ axonal neuropathy	ATX	exon 56	c.9004A>G	p.Ile3002Val	p. Ile2995Val	SR26	missense	c-het	30119932
119	Case 2	Korean	M	39	ATX+ autonomic dysfunction+ axonal neuropathy	ATX	exon 79	c.15123G>T	p.Glu5041Asp	p. Glu5112Asp	SR46	missense	c-het	30119932
120	1-III-1	Chinese	M	10	ATX+MND+MTD + arthrogryposis	MND	exon 118	c.21568C>T	p.Arg7190Ter	p.Arg7261Ter	SR63	nonsense	hom	in this tudy

121	1-III-2	Chinese	M	9	ATX+MND+low normal IQ	ATX	exon 118	c.21568C>T	p.Arg7190Ter	p.Arg7261Ter	SR63	nonsense	hom	in this study
122	2-II-2	Chinese	M	15	ATX+MND+cognitive impairment	Cognitive impairment	exon 95	c.17944C>T	p.Arg5982Ter	p.Arg6053Ter	SR53	nonsense	c-het	in this study
122	2-II-2	Chinese	M	15	ATX+MND+cognitive impairment	Cognitive impairment	exon 100	c.18684G>A	p.Trp6228Ter	p.Trp6299Ter	SR54-SR55	nonsense	c-het	in this stduy

Abbreviations: AAO = age at onset; Reference (PMID) = PMID of reference literature in Pubmed database; NA = data unavailable; M = male; F = female; Hom = homozygous; C-het = one of compound heterozygous; Het = reported as heterozygous and no evidence of compound heterozygous; ATX = ataxia; AMC = arthrogryposis multiplex congenital; EDMD = Emery-Dreifuss muscular dystrophy; CMD = congenital muscular dystrophy; DCM = dilated cardiomyopathy; AUT = autism; MTD = mental retardation; MND = motor neuron disorder. CH, a pair of N-terminal calponin homology domains; SR, spectrin repeats; KASH, C-terminal Klarsicht/ANC-1/Syne homology (KASH) domain.1[a] indicates that a total of 24 ataxia patients, but without detailed individual information.

Supplementary material_Table S4 Distribution of variants sites in reported *SYNE1* ataxia patients with motor neuron disorders

Variants sites of *SYNE1*	CH1	CH2	SR1	SR2	SR3	SR4	SR5	SR6	SR7	SR8	SR9	SR10	SR11	SR12	SR13
Number of Variants	0	5	0	0	0	1	0	1	0	0	0	0	0	0	1
Variants sites of *SYNE1*	SR14	SR15	SR16	SR17	SR18	SR19	SR20	SR21	SR22	SR23	SR24	SR25	SR26	SR27	SR28
Number of Variants	0	0	0	1	0	0	0	0	0	0	0	1	0	0	0
Variants sites of *SYNE1*	SR29	SR30	SR31	SR32	SR33	SR34	SR35	SR36	SR37	SR38	SR39	SR40	SR41	SR42	SR43
Number of Variants	0	1	0	1	0	0	0	1	0	0	1	2	0	0	1
Variants sites of *SYNE1*	SR44	SR45	SR46	SR47	SR48	SR49	SR50	SR51	SR52	SR52-53	SR53	SR54	SR54-55	SR55	SR56
Number of Variants	0	0	0	0	0	0	0	0	0	1	2	0	3	0	0
Variants sites of *SYNE1*	SR57	SR58	SR59	SR60	SR61	SR62	SR63	SR64	SR65	SR66	SR67	SR68	SR69	SR70	SR71
Number of Variants	1	1	2	0	2	1	6	0	1	0	0	1	0	2	0
Variants sites of *SYNE1*	SR71-72	SR72	SR73	SR74	KASH										
Number of Variants	1	0	0	1	0										

Abbreviations: CH, a pair of N-terminal calponin homology domains; SR, spectrin repeats; KASH, C-terminal Klarsicht/ANC-1/Syne homology (KASH) domain.
